# Iron overload causes a mild and transient increase in acute lung injury

**DOI:** 10.14814/phy2.14470

**Published:** 2020-06-29

**Authors:** Vida Zhang, Tomas Ganz, Elizabeta Nemeth, Airie Kim

**Affiliations:** ^1^ Department of Medicine David Geffen School of Medicine UCLA Los Angeles CA USA; ^2^ Department of Molecular and Medical Pharmacology UCLA Los Angeles CA USA

**Keywords:** acute lung injury, ARDS, inflammation, iron overload

## Abstract

Recent studies have demonstrated a strong link between acute respiratory distress syndrome (ARDS) and the levels of iron and iron‐related proteins in the lungs. However, the role of iron overload in ARDS development has yet to be characterized. In this study, we compared the highly iron‐overloaded hepcidin knockout mice (HKO) to their iron‐sufficient wild‐type (WT) littermates in a model of sterile acute lung injury (ALI) induced by treatment with oropharyngeal (OP) LPS. There were no major differences in systemic inflammatory response or airway neutrophil infiltration between the two groups at the time of maximal injury (days 2 and 3) or during the recovery phase (day 7). Hepcidin knockout mice had transiently increased bronchoalveolar lavage fluid (BALF) protein and MPO activity in the lung and BALF on day 3, indicating worse vascular leakage and increased neutrophil activity, respectively. The increased ALI severity in iron‐overloaded mice may be a result of increased apoptosis of lung tissue, as evidenced by an increase in cleaved capsase‐3 protein in lung homogenates from HKO mice versus WT mice on day 3. Altogether, our data suggest that even severe iron overload has a relatively minor and transient effect in LPS‐induced ALI.

## Graphical abstract

We used a mouse model of severe iron overload in order to investigate the effects of iron overload in the development of acute lung injury. We found that our overloaded mice developed a mild and transient worsening of acute lung injury, potentially via apoptosis.

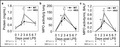

## 1 INTRODUCTION

Acute respiratory distress syndrome (ARDS) is a highly prevalent pathology with considerable associated morbidities and mortality. The etiologies of ARDS are varied, and include infectious pneumonia, sepsis, aspiration of gastric or oral contents, and major trauma (Matthay et al., 2019). Aside from treating the underlying pathologies, current therapies are primarily focused on supportive care and minimizing long‐term damage from the destructive effects of ARDS and mechanical ventilation. Despite advances in ARDS therapeutics, including low tidal volume ventilation (Brower et al., 2000), neuromuscular agents (Papazian et al., 2010), and prone positioning (Sud et al., 2010), patient outcomes remain unacceptably poor. The recent LUNG SAFE (Large Observational Study to Understand the Global Impact of Severe Acute Respiratory Failure) was a multicenter international prospective study of ARDS patients in Intensive Care Units (ICUs) in 2014 (Bellani et al., (2016)). Of the 29,144 patients admitted to participating ICUs, >10% fulfilled ARDS criteria, and in‐hospital mortality ranged from 35% to 46% depending on disease severity. Among those patients that survive ARDS, multiple studies have documented the long‐term detrimental effects on health‐related quality of life, with a high incidence of posttraumatic stress disorder (PTSD) symptoms (Davidson, Caldwell, Curtis, Hudson, & Steinberg, 1999; Schelling et al., 1998). Coupled with the staggering costs associated with an episode of ARDS, estimated at $170,000 for the hospitalization alone (Bice, Cox, & Carson, 2013), this disease process is a large public health concern that warrants persistent investigation into its pathophysiologic mechanisms.

One recent area of ARDS research is the role of iron in the development of ARDS (Zhang, Nemeth, and Kim (2019)). While iron is an essential trace mineral that is required for oxygen carrying capacity, DNA synthesis and repair, and cellular energy metabolism (Ganz & Nemeth, 2006), an excess of iron has deleterious effects for the host by leading to cellular toxicity via iron‐generated oxyradicals and peroxidation of lipid membranes (Ramm & Ruddell, 2005). Because of their direct exposure to the environment's particulate matter and high oxygen levels, the lungs are uniquely vulnerable to iron‐induced oxidative damage via Fenton's reaction (Dixon & Stockwell, 2014). The pathophysiology of ARDS is known to be mediated by the release of free radicals and reactive oxygen species and their injurious effects on endothelial integrity (Chabot, Mitchell, Gutteridge, & Evans, 1998).

While the clinical literature indicates a strong relationship between ARDS severity and alterations in the levels of iron and iron‐related proteins (Connelly et al., 1997; Ghio et al., 2003; Sharkey et al., 1999), there has been no systematic experimental investigation of the effects of iron overload on the development of ARDS in a mouse model. We examined the effect of severe iron overload on LPS‐induced acute inflammatory lung injury in a mouse model. Extreme iron overload was induced by the combination of a high‐iron diet and the deficiency of the iron‐regulatory hormone hepcidin (the whole‐body *Hamp1* knockout mouse) (Lesbordes‐Brion et al., 2006). Hepcidin deficiency causes unregulated intestinal iron absorption, making mice highly susceptible to extreme tissue iron overload from high‐iron diet and increased levels of circulating iron. Hepcidin knockout (HKO) mice were previously shown to develop pulmonary iron overload (Deschemin, Mathieu, Zumerle, Peyssonnaux, & Vaulont, 2017; Neves et al., 2017). This study aimed to detect the potentially detrimental effects of iron overload on the development of ARDS, by comparing HKO mice fed with high‐iron diet to their WT littermates on normal iron diet after both groups were challenged with LPS aspiration.

## 2 METHODS

### 2.1 Animal models

All animal studies were approved by the University of California Los Angeles (UCLA) Office of Animal Research Oversight. Hepcidin knockout mice were originally provided to our laboratory by Dr. Sophie Vaulont (Lesbordes‐Brion et al., 2006) and were backcrossed onto the C57BL/6J background (Ramos et al., 2012). Under specific pathogen‐free conditions, *Hamp1 ± *mice were bred to produce *Hamp1‐/‐* (HKO) mice and wild‐type (WT) *Hamp1+/+* mice for littermate controls. Seven‐ to twelve‐week‐old age‐ and sex‐matched male and female mice were used in experiments. Hepcidin knockout mice were placed on a high‐iron diet (5,000 ppm iron; Teklad diet TD.140464, Envigo) at 3 weeks of age for 3–4 weeks to augment iron loading, then placed on standard chow diet. WT mice were maintained on standard chow.

To induce acute lung injury ALI, mice were treated with 15 mg/kg LPS in saline through oropharyngeal (OP) aspiration, which was modified from De Vooght, et al. (De Vooght et al., 2009) In brief, mice are anesthetized with an intraperitoneal injection of ketamine/xylazine (Sigma‐Aldrich), then suspended on a wire by the front incisors. After placement of a nose clip, the tongue is gently pulled out of the mouth and to the side using forceps. The LPS solution is then pipetted into the posterior oropharyngeal space, and the nose and tongue released 5 s after the solution is aspirated into the lungs. The dose of 15 mg/kg LPS was used after a titration study for the lowest dose necessary to consistently yield evaluable ALI parameters. Control mice were not administered any saline by OP route because our goal was for the control group to not be exposed to any inflammation. We used LPS‐injected mice as a model of generalized inflammation, rather than to investigate the specific effects of LPS or the TLR4 pathway on lung injury.

Mice were euthanized 2, 3, and 7 days after LPS treatment, and bronchoalveolar lavage fluid (BALF) was obtained by lavaging the lungs three times with 0.8 ml of cold phosphate‐buffered saline (PBS). Lungs were perfused with 5 ml of cold PBS via the right ventricle, then lungs and liver were harvested and snap frozen in liquid nitrogen for analysis. Bronchoalveolar lavage fluid cell number was counted using a hemacytometer, and a cytocentrifuge (CytoSpin3 Cytocentrifuge; Shandon) was used to make slides. Cell differential staining was performed using the Hema 3^TM^ Fixative and Solutions (Fisher), and the percentages of different immune cell populations were counted manually. Bronchoalveolar lavage fluid protein was measured using the Pierce BCA Protein Assay Kit (Thermo Fisher).

### 2.2 Quantitative PCR

Tissue RNA was extracted via TRIzol (Thermo Fisher), and cDNA was synthesized using the iScript cDNA Synthesis Kit (BioRad). Gene transcript levels were quantified in duplicate by SsoAdvanced Universal SYBR Green Supermix (BioRad) using a CFX Connect or CFX96 Touch Real‐Time PCR Detection System (BioRad). mRNA expression was calculated using the ΔCT method normalized to hypoxanthine guanine phosphoribosyl transferase (Hprt) expression levels. Primers are listed in Table 1.

**TABLE 1:** Primers for RT‐PCR

	ForwardPrimer (5′ –> 3′)	Reverse Primer(5′ –> 3′)
Mouse Hprt	CTG GTT AAG CAG TAC AGC CCC	CGA GAG GTC CTT TTC ACC AGC
Mouse Saa‐1	TGA CCA GGA AGC CAA CAG	GTA GGA AGA CCA GAC C
Mouse Il6	CTC TGC AA GAGA CTT CCA TCC	CGT GGT TGT CAC CAG CAT CA
Mouse Tnf	AAT GGC CTC CCT CTC ATC AG	GCT ACG ACGTGG GCT ACA GG
Mouse Nqo1	CAC GGG GAC ATG AAC GTC AT	GGA GTG TGG CCA ATG CTG TA
Mouse Il1b	CCT TCC AGG ATG AGG ACA TGA	TGA GTC ACA GAG GAT GGG CTC
Mouse Hamp	CCT ATC TCC ATC AAC AGA TG	AAC AGA TAC CAC ACT GGG AA

### 2.3 Iron measurements

After lung and liver tissues were ground up in liquid nitrogen in order to homogenize the samples, lung and liver non‐heme iron concentrations were determined by a colorimetric assay for iron quantification (Sekisui Diagnostics). ~40 mg tissue was weighed and digested in a fixed volume of acid (3M HCl, 10% trichloroacetic acid) for 1 hr at 95°C. Samples were centrifuged to clear the insoluble material, then iron levels in the supernatant were quantified according to manufacturer instructions.

### 2.4 MPO assay

A quantity of ~10 mg of ground lung tissue was weighed and homogenized in 50 mM potassium phosphate solution, pH 6.0. The solution was centrifuged, then the pellet resuspended in 0.5% CETAB in Cell‐Based Assay Buffer (Cayman #10009322). After sonication and incubation, MPO activity in the supernatant was measured using the colorimetric neutrophil myeloperoxidase activity assay kit (Cayman #600620). BALF MPO activity was measured directly with the assay kit. MPO activity was measured as the rate of change of absorbance over time.

### 2.5 Western blot

Ground lung tissue was homogenized in RIPA buffer containing protease and phosphatase inhibitors. Protein concentration in lung lysates was determined by a BCA assay (ThermoFisher Pierce, 23225), and then lysates were denatured by SDS and boiling. The following antibodies were used for western blot: anti‐mouse cleaved caspase 1 (Cell Signaling #89332), anti‐mouse cleaved caspase 3 (Cell Signaling #9661), HRP‐conjugated anti‐beta actin (Sigma A3854), HRP‐conjugated anti‐rabbit IgG (ThermoFisher #31462).

### 2.6 Statistics

SigmaPlot (Systat Software) was used for all statistical analyses. Normally distributed data were analyzed using Student's *t* test, and not normally distributed data were analyzed using the nonparametric Mann–Whitney rank sum test. Multivariate analyses of the effects of genotype and time point on outcome were performed using a two‐way ANOVA. *p* < .05 was considered statistically significant.

## 3 RESULTS

### 3.1 Systemic inflammatory response to LPS‐induced ALI is not affected by iron overload

In order to uncover any potential phenotypic difference in iron‐sufficient (regular chow) versus severely iron‐overloaded mice, we further iron‐loaded our HKO mice with a high‐iron (5,000 ppm Fe) diet starting at weaning for 3–4 weeks before returning them to regular chow. WT littermates were maintained on standard chow throughout the study as iron‐sufficient controls. After LPS administration on day 0, mice were euthanized and analyzed on days 2, 3, and 7, which represent the time points of maximum lung injury (days 2 and 3) as well as recovery (day 7). By analyzing mice during significant injury and during recovery, we aimed to test the hypothesis that iron overload could affect either the development of lung injury or the subsequent recovery.

**FIGURE 1:**
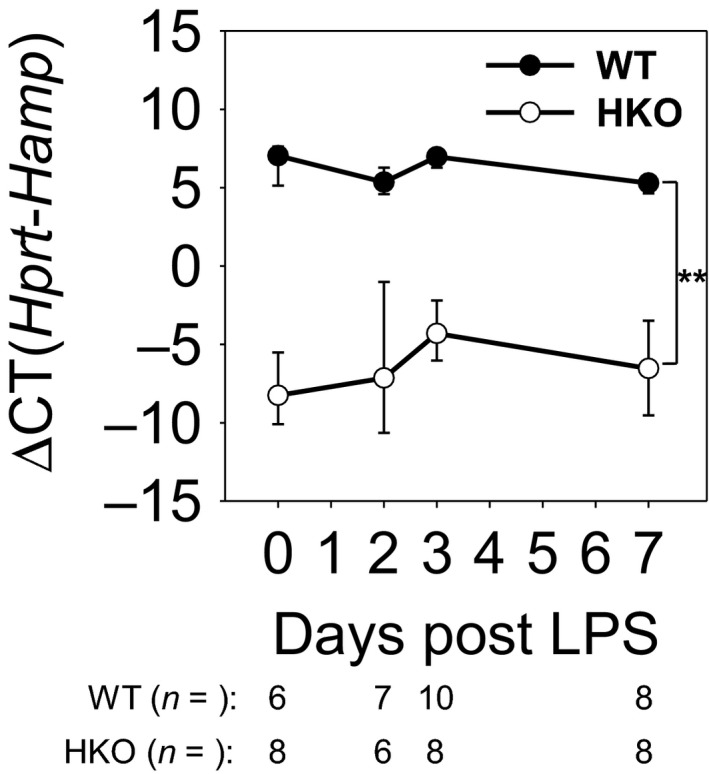
Hepcidin mRNA levels confirm knockout of hepcidin. WT and HKO mice were treated LPS OP, then analyzed on days 2, 3, and 7. Untreated WT and HKO mice were used as day 0 samples. Liver *Hamp* mRNA levels confirm knockout of hepcidin. Graph depicts mean ± *SD*; **p < .001 by a two‐way ANOVA for comparison of genotypes

**FIGURE 2:**
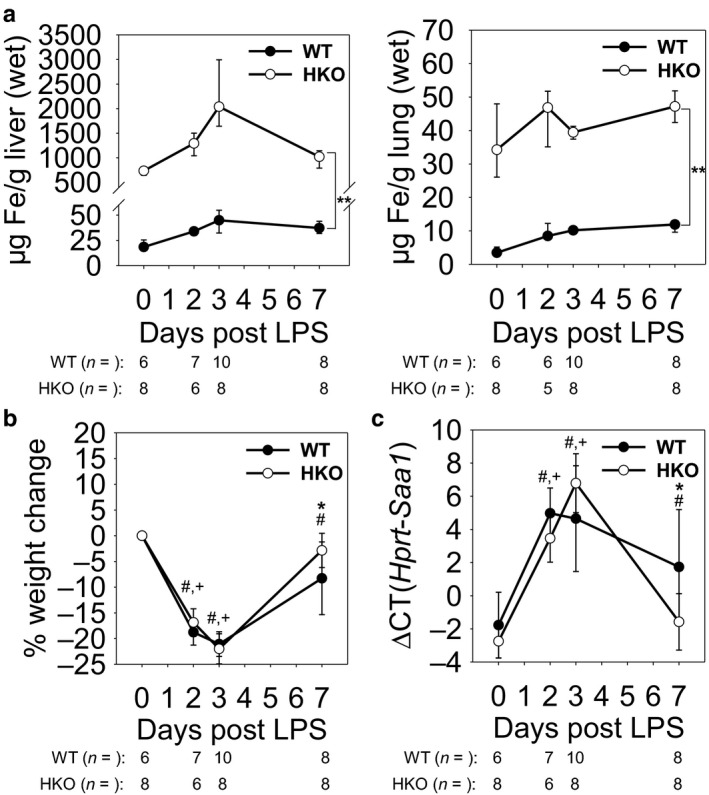
Systemic response to LPS aspiration is unaffected by iron overload. WT and HKO mice were treated with LPS OP, then analyzed on days 2, 3, and 7. Untreated WT and HKO mice were used as day 0 samples. (a) Non‐heme iron levels in *(left)* liver and *(right)* lung tissues confirm iron overload in HKO mice on high‐iron diet. (b and c) There was no significant difference in whole‐body response to LPS between WT and HKO mice as seen by (b) weight loss or (c) liver *Saa1* mRNA levels during peak inflammation. Graphs depict (a) median ± 25th/75th percentile or (b,c) mean ± *SD*; *p* < .05 by a two‐way ANOVA for comparison of WT (#) and HKO (+) mice versus day 0, *p < .05 and **p < .001 by a two‐way ANOVA for comparison of genotypes

Hepcidin deletion was confirmed by qRT‐PCR (Figure 1), and the two mouse groups were characterized by liver and lung non‐heme iron measurements (Figure 2a). As expected, HKO mice on high‐iron diet developed severe iron overload, with liver and lung iron levels 40‐fold and 10‐fold higher than those of iron‐sufficient mice, respectively, providing a stringent test of the hypothesis.

Systemic inflammatory response was assessed by monitoring weight loss and measuring levels of SAA‐1, which is induced by proinflammatory cytokines and is an accepted marker of IL‐6 activity (Hagihara et al., 2004). Mice in both groups showed evidence of systemic inflammation with significant weight loss of >20% from baseline by day 3 (Figure 2b). As expected, mice that were analyzed on day 7 had recovered more than half of their lost weight, with <10% weight loss from baseline. There was no significant difference in weight loss between the two groups at any time point, and there were no incidences of mortality in either mouse group throughout the course of the experiment (data not shown). *Saa1* mRNA levels similarly had peaked by days 2 and 3, with 32‐ and 128‐fold increases compared to baseline for WT and HKO mice, respectively (Figure 2c). Similar to the weight loss pattern, *Saa1* levels had largely recovered by day 7, and there was no significant difference between the two mouse groups during peak inflammation, days 2 and 3. Although there was a statistically significant difference in the *Saa1* mRNA levels on day 7, the difference is of questionable physiologic significance during this time of mostly resolved inflammation. Thus, we were able to establish that despite severe iron overloading, the systemic inflammatory response to OP LPS was similar in the iron‐sufficient and iron‐overloaded groups during both active inflammation and recovery.

### 3.2 Inflammatory cell infiltration of the airway is similar in iron‐sufficient and iron‐overloaded mice in the LPS model of ALI

**FIGURE 3:**
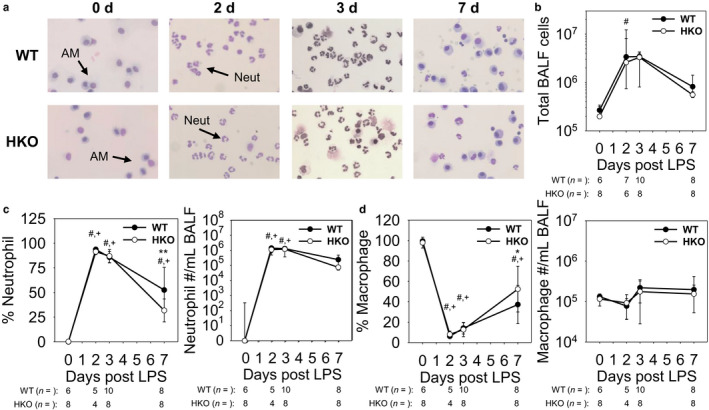
LPS induces airway neutrophil infiltration similarly in WT and HKO mice. (a) Representative images of BALF cell cytospins show an evolving leukocyte population. Neut: neutrophil; AM: alveolar macrophages. (b) Increased cell count and (c) % neutrophil and neutrophil count in BALF reflect neutrophil infiltration during the early and recovery phases of inflammation. (d) Though % macrophage in BALF decreases due to influx of neutrophils, absolute macrophage count in BALF remains similar throughout the course of inflammation. Graphs depict (b,c) median ± 25th/75th percentile or (c,d) mean ± *SD*; *p* < .05 by a two‐way ANOVA for comparison of WT (#) and HKO (+) mice versus day 0, *p < .05 and **p < .001 by a two‐way ANOVA for comparison of genotypes

The characteristics of BALF leukocyte populations at each time point were evaluated to determine the degree of acute airway inflammation for each mouse group. BALF was centrifuged immediately after harvesting, and the respective cell pellets were characterized by cell counting and microscopic analysis via differential staining of cytospin slides (Figure 3a). The total number of BALF leukocytes per mouse peaked by day 2 at 3.4 × 10^6^and 2.6 × 10^6^for WT and HKO mice, respectively, after which they began a gradual return to baseline levels (Figure 3b). As expected, baseline uninflamed mouse airway immune cells were essentially all macrophages with very few neutrophils, but acutely inflamed airway immune cell populations were made up of > 90% neutrophils on days 2 and 3 (Figure 3c,d). During the recovery phase of the inflammation, mouse BALF leukocyte subpopulations began to return to their baseline composition (Alber, Howie, Wallace, & Hirani, 2012). Of note, the differences in total leukocyte numbers were primarily driven by neutrophil recruitment into the lungs. While macrophage percentages varied widely depending on the influx of neutrophils, absolute macrophage numbers remained relatively stable through the course of the inflammation (Figure 3d). In comparing the evolution of the leukocyte profile of iron‐sufficient versus iron‐overloaded mice, aside from a minor increase in % neutrophils and decrease in % macrophages in BALF from WT mice compared to HKO mice on day 7, we found that there was no significant difference in any of the parameters at any time point. However, one potential caveat is that the neutrophil population at peak inflammation already made up >90% of the total leukocyte population in iron‐sufficient WT mice, which may have masked any additional increase in neutrophil percentage in iron‐overloaded HKO mice.

### 3.3 Iron‐overloaded mice develop a mild and transient increase in ALI severity compared to iron‐sufficient mice

**FIGURE 4:**
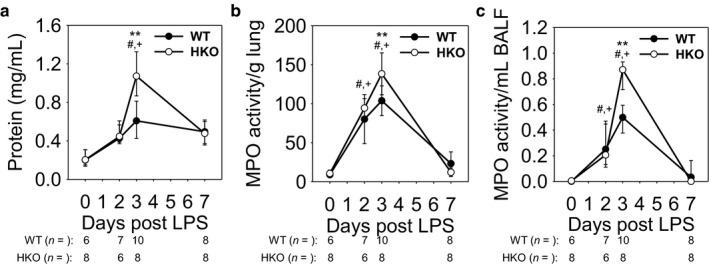
Iron‐overloaded mice develop a mild and transient increase in ALI severity. (a) BALF protein concentration is increased in HKO mice compared to WT mice at 3 d. MPO activity in (b) lung tissue and (c) BALF indicate increased ALI in HKO mice compared to WT mice at 3 d. Graphs depict (a,c) median ± 25th/75^th^percentile or (b) mean ± *SD*; *p* < .05 by a two‐way ANOVA for comparison of WT (#) and HKO (+) mice versus day 0, **p < .001 by a two‐way ANOVA for comparison of genotypes

We measured BALF protein and MPO activity of the lung and BALF in order to assess and quantify the development of ALI. BALF protein levels indicated that vascular fluid leakage and pulmonary edema were most severe for both mouse groups at day 3, but with significantly increased BALF protein levels in iron‐overloaded mice (WT 0.6 mg/ml vs. HKO 1.1 mg/ml; *p* < .001) **(**Figure 4a). Similarly, MPO activity levels showed that neutrophil activity was highest at day 3 for both groups, and iron‐overloaded mice had significantly higher activity levels in both lung tissue (Figure 4b) and BALF (Figure 4c) at the same time point (WT lung MPO 104 vs. HKO lung MPO 138; *p* < .001) (WT BALF MPO 0.5 vs. HKO BALF MPO 0.9; *p* < .001). Interestingly, MPO activity in BALF was higher in HKO compared to WT mice despite comparable number of neutrophils in the two groups. This may reflect increased secretion of MPO from neutrophils in the HKO group. ALI parameters by day 7 indicated substantial recovery for both groups, with no difference between iron‐sufficient and iron‐overloaded mice. Thus, severely iron‐overloaded mice developed worse vascular leakage and lung injury after LPS aspiration, but the effect was transient and did not affect recovery.

### 3.4 The transiently more severe ALI phenotype in iron‐overloaded mice may be caused by increased apoptosis, and not by changes in local cytokine production, ROS generation, or pyroptosis

**FIGURE 5:**
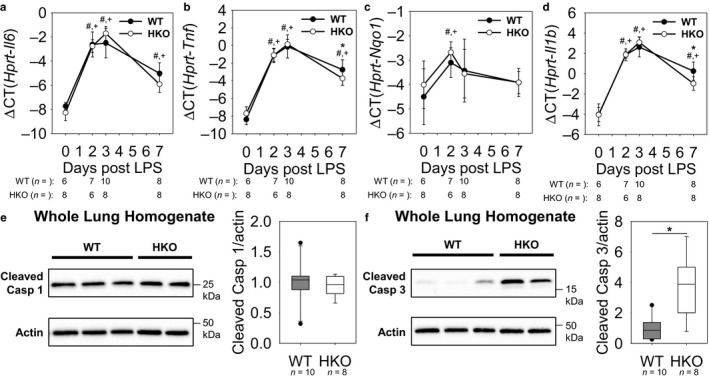
More severe ALI in iron‐overloaded mice may be caused by increased apoptotic cell death. Lung (a) *Il6* mRNA and (b) *Tnf* mRNA show similar induction of lung inflammation between WT and HKO mice. (c) *Nqo1* mRNA levels indicate similar ROS generation between WT and HKO mice. Similar lung (d) *Il1b* mRNA levels and (e) cleaved caspase‐1 levels at 3d between the genotypes indicate that there is no difference in pyroptosis. (f) Cleaved caspase‐3 is increased in HKO mice compared to WT mice at 3 d, suggesting increased apoptosis. Graphs depict (a‐d) mean ± *SD* or (e, f) median ± 25th/75th percentile; *p* < .05 by a two way ANOVA for comparison of WT (#) and HKO (+) mice versus day 0, *p < .05 by (b, d) a two‐way ANOVA for comparison of genotypes or (f) Mann Whitney rank‐sum test for comparison of genotypes

In order to identify potential causes of the worse ALI that occurs with iron overload, we investigated established mechanisms that contribute to the development of ALI: lung inflammatory cytokines (Cross & Matthay, 2011), ROS generation (Kellner et al., 2017), pyroptosis (Li, Ren, Jiang, & Zhu, 2018), and apoptosis (Lu, Harrington, & Rounds, 2005). As expected, lung levels of *Il6* and *Tnf* mRNA peaked near days 2 and 3 and showed partial recovery by day 7 (Figure 5a,b). However, there was no difference in the levels of these proinflammatory cytokines between the two groups of mice on days 2 and 3. Similarly, lung *Nqo1* mRNA indicated that ROS generation was highest on day 2, with a significant decrease in ROS by day 7 (Figure 5c), but there were no significant differences between the mouse groups at any time point. Lung *Il1b* mRNA and cleaved caspase‐1 protein were measured as markers of pyroptosis, and showed no significant difference with iron overload on days 2 and 3, and day 3, respectively (Figure 5d,e). Of note, there were subtle differences between WT and HKO mice in the levels of *Tnf* and *II1b* mRNA on day 7, but these were not associated with any differences in ALI parameters. However, cleaved caspase‐3 protein was increased in whole lung of HKO mice versus WT mice on day 3, indicating that iron‐overloaded mice had increased apoptosis as compared to iron‐sufficient mice (Figure 5f). Thus, our data suggest increased apoptosis in iron‐overloaded mice that could exacerbate ALI but these effects are transient.

## 4 DISCUSSION

ALI, clinically known as ARDS, is an acute inflammatory process characterized by neutrophil infiltration, increased vascular permeability, and diffuse alveolar damage (Johnson & Matthay, 2010). Despite its high incidence, conservatively estimated at 150,000 new cases/year in the United States (Reynolds et al., 1998), and high cost to the patient and to society, therapeutic options are limited and there are large gaps in our understanding of this disease process. One underexplored but promising area of research is the role of iron in the pathophysiology of ARDS. Because of the strong link between iron and oxidative stress with endothelial injury, the mechanistic study of iron in lung injury has received early attention in recent literature. In addition, iron status is of particular importance in the ICU, where approximately one‐third of patients receive a blood transfusion and effectively a large iron load (Lelubre & Vincent, 2011). In this study, we have performed a systematic examination of ALI after OP LPS administration, comparing iron‐sufficient versus severely iron‐overloaded mice.

Multiple clinical studies have demonstrated the relevance of iron in the development of ARDS, showing strong correlations with iron and iron‐related proteins and the presence or severity of ARDS. Ghio, et al. showed that ARDS patients had increased concentrations of BALF total iron and non‐heme iron as compared to healthy controls (Ghio et al., 2003). Other iron‐related proteins, including hemoglobin, transferrin, transferrin receptor, lactoferrin, and ferritin were also all elevated in the BALF. Whether these changes in BALF iron and iron‐related proteins have a causal effect on lung injury or are simply a byproduct of increased vascular permeability is pending further study. Serum ferritin has also been investigated as a predictor of ARDS, with the caveat that ferritin is not only a marker of body iron stores, but also a known acute phase reactant. One clinical study reported serum ferritin to predict the development of ARDS with high sensitivity and specificity (Connelly et al., 1997). A second clinical study confirmed the predictive value of ferritin for ARDS in a more homogeneous group of patients who were at risk for trauma‐related ARDS, (Sharkey et al., 1999) and found no correlation of ferritin with other clinical outcomes, including the severity of hypoxia, time of invasive ventilation, and mortality.

Another clinically important question in iron management during ARDS is whether there is an infectious component. Increased iron status has been associated with multiple infectious complications, including increased incidence of bacteremia with hemodialysis (Jean et al., 2002), the development of active tuberculosis in a high‐risk population (Gangaidzo et al., 2001), and activation of preexisting malaria (Murray, Murray, Murray, & Murray, 1978). As pneumonia and other infectious processes often occur concurrently with ARDS, either as the primary cause or a secondary complication, the role of iron in infection virulence is clinically relevant for ARDS patients. Several animal studies have specifically examined the contribution of iron to disease severity. Michels, et al. found that hepcidin‐mediated iron sequestration was protective in a mouse model of *Klebsiella pneumoniae* pneumonia, with hepcidin agonist treatment leading to decreased bacterial burden and improved survival (Michels et al., 2017). Another animal study infused commercial human haptoglobin into dog models of *Staphylococcus aureus* pneumonia, and found that treated dogs had increased clearance of cell‐free hemoglobin with lower circulating iron, less injury, and increased survival (Remy et al., 2018). The authors concluded that the reduced iron levels limited nutrient availability to microbes, as well as decreased the ability for iron to cause extravascular oxidative tissue injury. Another group used cecal ligation and puncture (CLP) surgery as a model of polymicrobial sepsis induced pneumonia and ALI. The knockdown of airway epithelial cell hepcidin using adenovirus‐mediated short hairpin RNA increased the severity of sepsis‐induced lung injury, bacterial burden, and mortality (Chen et al., 2014).

In contrast to the abundance of animal studies examining the interplay of iron and infections, there has been a paucity of similar investigation into the role of iron in sterile ALI. One study compared WT to *Hfe‐/‐* mice, a model of moderate iron loading, after an intratracheal administration of LPS (Benesova et al., 2012). The study found attenuated neutrophil recruitment to bronchoalveolar space in *Hfe‐/‐* mice with no difference in circulating neutrophil numbers. *Hfe‐/‐* mice also had altered expression of a subset of cytokines, suggesting that iron loading could worsen inflammatory injury. Another study (Deschemin et al., 2017) compared the response of WT and HKO mice to an acute LPS stimulus (6 hr), but did not find differences in lung chemokine expression or MPO activity at this early time point. To elucidate the mechanism of iron involvement in the development of ARDS, we must be careful to separate the direct contributions of iron to lung injury by ROS generation versus bacterial virulence with metal nutrient availability. In order to examine this question, we used an LPS mouse model to mimic the lung injury from a gram‐negative rod pathogen, independently of any effects of microbial proliferation. To our knowledge, our current study is the first animal study to systematically investigate the contribution of iron overload to the pathophysiology of sterile ALI.

In designing this study to test the effects of iron loading in the development of ALI, we chose to compare mouse groups with extreme differences in iron loading, that is, WT mice fed normal chow versus HKO mice fed high‐iron diet. The extensive literature using HKO mice does not document an iron‐independent effect of hepcidin deletion, thus allowing us to use these mice as a pure model of iron overload (Khorramian et al., 2017; Kim et al., 2014; Lesbordes‐Brion et al., 2006; Ramos et al., 2012). However, a potential limitation of this study is that it excluded groups of animals with intermediate iron loading, that is, WT mice fed high‐iron diet or HKO mice fed regular chow. While the inclusion of these intermediate groups may have provided more subtle differences in phenotypes, the lack of a major difference in the mouse groups with extreme differences in iron status did not allow justification for the addition of more intermediate iron loaded groups.

Although we used a mouse model of extreme iron overload in order to magnify any phenotype, we saw only a mild and transient increase in ALI severity with no impact on recovery. This suggests that even severe systemic iron overload may not appreciably affect the clinical course and outcome of acute lung injury. To examine the mechanism of the mild increase in ALI in our iron‐overloaded mice, we considered the potential involvement of known contributors to ARDS development: inflammatory cytokines (Matthay et al., 2019), ROS generation (Tasaka, Amaya, Hashimoto, & Ishizaka, 2008), apoptosis (Matute‐Bello & Martin, 2003), and pyroptosis (Cheng et al., 2017). Interestingly, we found a difference only in the degree of apoptosis between iron‐sufficient and iron‐overloaded mice, with no major differences in our measurements of local cytokines, ROS generation, or pyroptosis. Thus, our study suggests that apoptosis could be the pathway of the minor iron effects in ALI. Although previous literature has shown that apoptosis in ARDS is most relevant in epithelial cells (Galani et al., 2010), further investigation is necessary to confirm the cell type that is the site of increased apoptosis in the context of iron overload.

Our mouse model of iron overload showed that even severe iron excess plays a modest role in lung injury severity in the absence of infection. Further study is necessary to examine the effects of iron status in patients with ARDS, as well as to elucidate potential contributory mechanisms.

## CONFLICT OF INTEREST

T.G. and E.N. are shareholders and scientific advisors of Intrinsic LifeSciences and Silarus Therapeutics, and consultants for Ionis Pharmaceuticals, Protagonist, Keryx Pharmaceuticals, La Jolla Pharma, Vifor, Akebia (T.G.), and Gilead (T.G.). Neither A.K. nor V.Z. have any conflicts of interest, financial, or otherwise, to disclose.

## AUTHOR CONTRIBUTIONS

T.G., E.N., and A.K. conceived and designed the experiments; V.Z. and A.K. performed experiments and prepared figures and manuscript; V.Z., T.G., E.N., and A.K. analyzed the data and approved final version of manuscript.
